# Frequency, clinical characteristics, risks, and outcomes of Paradoxical upgrading reactions during anti-tuberculosis treatment in tuberculous lymphadenitis

**DOI:** 10.12669/pjms.36.ICON-Suppl.1711

**Published:** 2020-01

**Authors:** Samreen Sarfaraz, Sundus Iftikhar, Naseem Salahuddin

**Affiliations:** 1 Samreen Sarfaraz, MRCP. Infectious Disease Department, The Indus Hospital, Karachi, Pakistan; 2 Sundus Iftikhar, MPhil. Indus Hospital Research Center, The Indus Hospital, Karachi, Pakistan; 3 Naseem Salahuddin, D.A.B.I.M. Infectious Disease Department, The Indus Hospital, Karachi, Pakistan

**Keywords:** Paradoxical upgrading reactions, Tuberculous lymphadenitis, Anorexia, Caseous necrosis, AFB smear positivity

## Abstract

**Objectives:**

To investigate the clinical characteristics, risks and outcomes of Paradoxical upgrading reactions (PUR) during anti-tuberculosis treatment (ATT) in superficial tuberculous lymphadenitis (TBLA).

**Methods:**

In this nested case-control study, all patients diagnosed with TBLA based on combinations of histopathology, acid-fast bacilli (AFB) microscopy, AFB culture, and GeneXpert, between February 2013 and April 2016, were enrolled. Standard ATT was given. Demographics, clinical characteristics, occurrence of PUR and outcome were recorded.

**Results:**

TBLA was diagnosed and treated in 189 patients. PUR developed in 33 (17%), of which 77% developed new inflamed glands, 20.6% had increased size and inflammation of pre-existing glands and 5.9% had superficial chest wall abscesses requiring aspiration. All responded to regular NSAIDs except one, where a steroid course was effective. No change in dose or duration of ATT was required. Presence of anorexia (OR; 95%CI: 2.6; 1.003-6.74), bilateral extensive lymphadenopathy (OR; 95%CI: 2.9; 1.1-7.5) and lymph node specimen positive for AFB (OR; 95%CI: 3.2; 1.04-10.1) were significantly associated with PUR.

**Conclusion:**

PUR is common in TBLA. It responded to NSAIDS and does not need any modification in ATT.

## INTRODUCTION

It has been long recognized that similar to leprosy, distinct immunological reactions may complicate the course of effective sterilizing anti-tuberculous therapy (ATT).^1^ The first described report of paradoxical worsening on effective ATT was in 1954.^2^ This phenomenon, termed paradoxical upgrading reaction (PUR), results in clinical or radiological deterioration of pre-existing tuberculous lesions or development of new lesions after an initial favourable response. Documented clinical presentations of PUR include development or expansion of intracranial tuberculomas,^3^ lymph node enlargement,^4^ worsening chest infiltrates,^5^ new onset pleural effusion or suppuration at different sites. PUR is unpredictable in its timing, severity or duration. It is usually mild and self-limiting but rarely respiratory failure and death have been described.^6^

Differentiating PUR from treatment failure, drug resistance or inflammation due to a concomitant infection may be diagnostically challenging but is important as PUR does not require ATT alteration. Failure to recognize PUR may result in unnecessary prolongation of ATT or injudicious escalation to second-line drugs. Most cases of PUR occur in complicated tuberculous lymphadenitis (TBLA) or intracranial tuberculosis^7^ and in both non-HIV^8^ and HIV infected patients.^1^ Whereas enough is known about immune constitution reactions in HIV patients, the literature on risk factors and clinical characteristics of PUR in HIV negative patients with tuberculosis is limited.^9^

As there was a scarcity of literature on PUR in our region, we undertook a study to report the frequency, clinical spectrum and risk factors of PUR in patients with TBLA, the most common forms of extrapulmonary tuberculosis. This data will help in educating physicians and other healthcare providers about PUR and distinguishing it from treatment failure, eventually improving patient management and TBLA outcomes in the local and global context.

## METHODS

In this nested case-control study, all confirmed or presumed TBLA patients from the Infectious Disease Clinic, The Indus Hospital (TIH), Karachi, Pakistan were enrolled after IRB approval as IRD_IRB_2013_01_002. Patients commenced and completed treatment over a 3-year period from February 2013 to April 2016. Confirmed TBLA was diagnosed if there was either mycobacterial growth on culture, or MTB positive on GenExpert or AFB on microscopy. A patient was labelled as presumed TB if there was no microbiological evidence of TBLA but suggestive histopathology.^10^ Histological patterns suggestive of TBLA were the presence of chronic granulomatous inflammation, acute necrotizing or suppurative inflammation or extensive caseous necrosis. All lymph node specimens were processed for histopathology, microscopy, GeneXpert and AFB culture using standard techniques.

PUR was defined as clinical or radiological worsening of pre-existing tuberculous lesions or development of new lesions following at least two weeks of appropriate ATT with initial clinical improvement. Before commencing ATT the site, size, number, and any tenderness of nodes was recorded. A baseline complete blood count (CBC), chest radiograph (CXR), and erythrocyte sedimentation rate (ESR) was performed. Standard first-line ATT with Isoniazid (H), Rifampicin (R), Ethambutol (E) and Pyrazinamide (Z) was prescribed for six months in new cases. In re-treatment cases, eight months of ATT was given with HREZ and Streptomycin (S) in the intensive phase and HRE in the continuation phase. Compliance with ATT and development of PUR was recorded on follow-up. Patients were observed once after two months of completion of ATT for relapse of disease or post-therapy PUR.

Data were analyzed using SPSS version 24.0. Mean (SD) or Median (IQR) was computed as appropriate for all the quantitative variables. Frequency and percentage were computed for all categorical variables. Independent sample t-test or Mann-Whitney U test was applied as appropriate to assess differences in quantitative variables between the case group (with PUR) and the control group (without PUR). Chi-square test or Fisher-Exact test were applied as appropriate to assess the association between the various categorical variables and the occurrence of PUR. Univariate and multivariable logistic regression was applied to assess risk factors significantly associated with PUR. Variables with p value less than 0.25 were included in the final multivariable analysis and backward likelihood ratio method was applied to build the final model. P-value<0.05 was considered statistically significant for the final model.

## RESULTS

**Fig.1:**
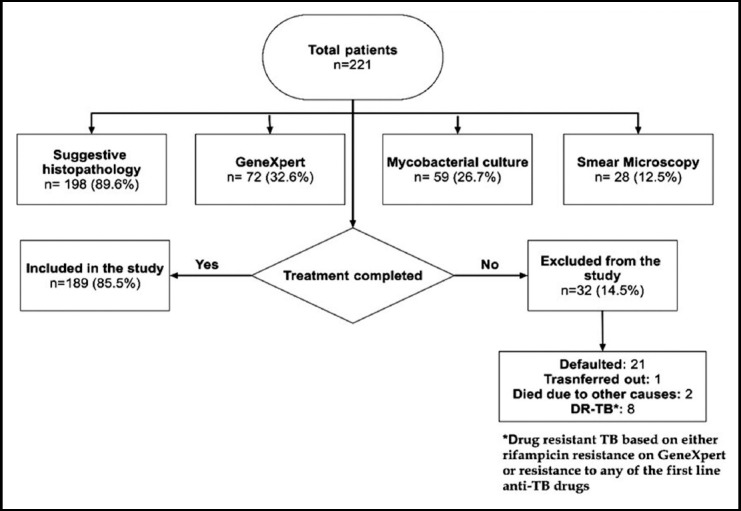
Flow chart of the study.

During a three-year study period, 221 patients were diagnosed as TBLA, of which 189 were followed on treatment with standard first-line ATT to record the clinical characteristics and risk factors associated with PUR development (Fig.1). In these 189 patients, PUR occurred in 33 (17.5%) patients during ATT (case group). In the case group, 22 (66.7%) were males. The median age was 22 (IQR=19.5-31.5) years (Table-I). Seven (21.2%) patients developed PUR in the first month, 22 (66.7%) in the 2nd to 4th month of the treatment and 4 (12.1%) in the last two months of treatment (Table-II).

**Table I:** Characteristics of the study participants according to IRIS.

	PUR n(%)		p-value

	not observed n=156	observed n=33
***Age in years; Median (IQR)***	24 (18-33.5)	22 (19.5-31.5)	0.744^ɬ^
***Gender***			
Female	140 (78.7)	28 (82.4)	0.626^‡^
Male	38 (21.3)	6 (17.6)	
***BMI; Median (IQR)***	19.4 (16.9-22.6)	20.3 (18.3-24.1)	0.274^ɬ^
***Duration of illness in days; Median (IQR)***	56 (21-121.7)	30.4 (14-168)	0.783^ɬ^
***Outcome***			
Treatment completed	156 (87.6)	33 (97.1)	0.860^†^
Lost to follow up	6 (3.4)	0 (0)	
Defaulted	11 (6.2)	1 (2.9)	
Died	2 (1.1)	0 (0)	
Treatment failure	2 (1.1)	0 (0)	
Transfer out	1 (0.6)	0 (0)
***Fever***			
No	72 (40.4)	9 (26.5)	0.124^‡^
Yes	106 (59.6)	25 (73.5)
***Appetite loss***			
No	103 (58.2)	13 (38.2)	0.032^*‡^
Yes	74 (41.8)	21 (61.8)
***Weight loss***			
No	85 (47.8)	12 (35.3)	0.182^‡^
Yes	93 (52.2)	22 (64.7)
***Persistent Cough***			
No	127 (72.6)	18 (54.5)	0.039^*‡^
Yes	48 (27.4)	15 (45.5)
***Diabetes***			
No	173 (97.7)	34 (100)	1.000^†^
Yes	4 (2.3)	0 (0)
***Hepatitis***			
No	173 (98.3)	34 (100)	1.000^†^
Yes	3 (1.7)	0 (0)
***HIV***			
No	171 (98.8)	33 (100)	1.000^†^
Yes	2 (1.2)	0 (0)
***Past history of TB***			
No	160 (89.9)	29 (85.3)	0.383^†^
Yes	18 (10.1)	5 (14.7)
***Node location***			
Cervical	136 (87.2)	28 (84.8)	0.131^†^
Axillary	1 (0.6)	0 (0.0)	
Inguinal	1 (0.6)	0 (0.0)	
Cervical and axillary	14 (9)	2 (6.1)	
Cervical and inguinal	1 (0.6)	3 (9.1)	
Cervical, axillary and inguinal	3 (1.9)	0 (0.0)	
Total	156 (100)	33 (100)	
***nodes size; Median (IQR)***	3.5 (2.9-4.5)	3.7 (3-4.6)	0.617^ɬ^
***Node type***			
Unilateral	137 (77)	21 (61.8)	0.062^‡^
Bilateral	41 (23)	13 (38.2)	
***Hb gm/dl; Mean ± SD***	11.4 ± 1.7	10.6 ± 1.9	0.107^ɫ^
***Total WBC count (cells/micro L); Median (IQR)***	7300 (5700-8400)	7400 (5900-8000)	0.677^ɬ^
***Total Lymphocyte count (cells/micro L); Median (IQR)***	1800 (1300-2200)	2.0 (1400-2300)	0.505^ɬ^
***ESR; Median (IQR)***	39.5 (22-68.25)	38 (24-83)	0.655^ɬ^
***X-ray finding***			
Mediastinal widening	42 (27.5)	6 (18.8)	0.087^†^
Hilar adenopathy	2 (1.3)	3 (9.4)	
Pleural Effusion	1 (0.7)	0 (0.0)	
Parenchymal infiltrates	26 (17)	8 (25.0)	
Normal	82 (53.6)	15 (46.9)
***Gene expert***			
Negative	103 (62.4)	15 (46.9)	0.100^‡^
Positive	62 (37.6)	17 (53.1)
***AFB smear***			
Negative	144 (88.3)	22 (66.7)	0.002^*†^
Positive	19 (11.7)	11 (33.3)
***AFB culture***			
Negative	108 (65.9)	21 (67.7)	0.839^‡^
Positive	56 (34.1)	10 (32.3)
***Histology***			
H1	5 (62.5)	3 (37.5)	0.088^†^
H2	28 (84.8)	5 (15.2)	
H3	50 (87.7)	7 (12.3)	
H4	14 (66.7)	7 (33.3)	
H5	33 (84.6)	6 (15.4)	
Nonspecific changes	10 (100)	0 (0.0)	

* P-value <0.05,

‡ Chi-square test,

† Fisher-exact test,

ɬ Mann-Whitney U test, ɫ Independent sample T-test.

H1: severe acute suppurative inflammation w/wo granuloma;

H2: acute necrotizing lymphadenitis inflammation w/wo granuloma;

H3: Chronic granulomatous inflammation; H4: Extensive Caseous necrosis w/wo granuloma;

H5: Reactive lymph hyperplasia w/wo granuloma.

**Table II:** Factors associated with PUR.

Univariate binary logistic regression			Multivariable binary logistic regression	

Variables	PUR observed		PUR observed	

	Crude OR	95% CI	adjusted OR	95% CI
Age in years	0.99	0.95-1.02	-	-
Hb	0.8	0.58-1.1	-	-
***Gender***				
Female	1.16	0.44-3.05	-	-
Male	ref		-	-
Fever	2.1	0.9-4.7	-	-
Cough	2.6	1.2-5.7	-	-
Appetite loss	2.1	0.97-45	2.6	1.003-6.74
Weight loss	1.75	0.81-3.8		
***Node type***				
Bilateral	2.1	0.92-4.6	2.9	1.1-7.5
Unilateral	ref		ref	
***AFB smear***				
Positive	2.9	1.2-7.2	3.24	1.04-10.1
Negative	ref		ref	
***Histology***				
H1	4.3	0.83-22	2.1	0.29-14.9
H2	1.3	0.37-4.4	0.76	0.2-2.9
H3	ref		ref	
H4	3.4	1.1-1.9	3.1	0.83-11.47
H5	1.3	0.4-4.2	1.1	0.31-3.7
Nonspecific changes	-	-	-	-

Reference category: PUR not detected, Logistics regression.

PUR developed in the form of emergence of new inflamed nodes in 24 (72.7%) patients; enlargement, suppuration or spontaneous rupture with sinus formation in affected tuberculous nodes in seven (21.2%) patients; and formation of superficial chest wall abscesses in two (6%) patients. PUR was successfully treated with administration of NSAIDs for 2-3 weeks in 30 (90.9%) patients, a short course of steroids in one patient after failed response to NSAIDS and simple aspiration of purulent fluid in the two patients with superficial chest wall abscesses. No change in ATT dose or duration was made. All patients successfully completed treatment with resolution or regression of glands. No cases relapsed or exhibited clinical features of post-therapy PUR two months after ATT completion. No significant difference was observed in age, gender distribution, body mass index and underlying comorbidity in the case group as compared to the control group (Table-I). There was one HIV positive patient with TBLA in the cohort but he did not develop a PUR on ATT.

On univariate analysis, the presence of persistent cough, positive AFB smear of affected lymph gland and extensive caseous necrosis, were found to be significantly associated with PUR with a 2.6 (95% CI: 1.2-5.7), 2.9 (1.2-7.2) and 3.4 (1.1-1.9) times higher odds of developing PUR respectively. On multivariable analysis presence of anorexia (OR; 95%CI: 2.6; 1.003-6.74), bilateral extensive lymphadenopathy (OR; 95%CI: 2.9; 1.1-7.5) and AFB positive lymph node (OR; 95%CI: 3.2; 1.04-10.1) were found to be significantly associated with PUR adjusting for histopathology (Table-II).

## DISCUSSION

PUR is an abnormal, excessive immune reaction to tuberculoproteins and other cell wall antigens of live or dead mycobacteria whose pathogenesis is poorly understood. A large bacillary load, host genetic susceptibility, immune reconstitution following effective ATT leading to recognition of antigens released in the mycobacterial killing are postulated as possible mechanisms.^11^ Consistent with previous studies on TBLA in HIV uninfected patients,^8^,^9^,^12^ PUR was observed in 17.5% of our patients, none of whom gave a history of HIV infection. The literature reports a variable median time for PUR development from 21-56 days after commencement of ATT.^13^ Sixty-six percent of our patients developed PUR between the second and fourth month of therapy but around 12% exhibited the reaction in the last two months, highlighting onset variability in our group. Post-therapy PUR is also well documented^14^ and may occur up to 22 months after stopping ATT.^4^ These are very difficult to differentiate from relapse, necessitating re-biopsy and re-treatment of affected nodes. However, no case of post-treatment PUR or relapse was observed in our study in the two months following ATT completion. Follow-up beyond this period to capture late reactions was beyond the scope of our study, so we may have missed some late post-therapy PURs.

Making a diagnosis of PUR is difficult as no pathognomonic clinical features or specific tests can reliably establish or exclude the diagnosis^11^ and differentials of treatment failure due to noncompliance, malabsorption, drug-resistant TB or an alternate diagnosis e.g. malignancy or another bacterial or fungal infection need to be excluded.^15^ However, an initial clinical improvement followed by paradoxical worsening is a consistent feature. In our study, compliance was ensured by questioning on each follow-up visit. Patients labeled as PUR had initial regression in nodal size as evidenced by objective measurement and experienced mild to moderate deterioration only which responded well to 2-3 weeks of NSAIDS, therefore no additional tests were needed to exclude drug resistance or an alternate diagnosis, nor was a change in ATT warranted. This is in contrast to studies where physicians tended to prolong ATT to 9-12 months in patients experiencing PUR.^9^ There is no consensus on the optimum adjunctive therapy needed to control PUR. Cho et al reported 54 patients with PUR from a total of 235 with TBLA, of which 56% had spontaneous resolution with no therapy, 39% needed re-biopsy, 27% had nodal excision, 19% needed aspiration and 4% responded to steroids,^9^ Irrespective of the treatment received by the patients, PUR in TBLA is generally self-limiting and resolves without serious sequelae, in around two months.^11^ We had an excellent response with NSAIDS, with inflammation subsiding in 3-4 weeks and a two week course of oral steroids was used only in one patient whose PUR failed to abate with NSAIDs. This is consistent with other studies where steroids have shown to stabilize pro-inflammatory cytokines in intracranial tuberculomas^3^ and only rarely needed in TBLA.^8^,^9^ Only two patients with superficial chest wall abscesses required aspiration, consistent with other reported cases of soft tissue abscesses and pleural effusions.^16^

Interestingly, patients with positive AFB on initial diagnostic smear were 3.24 times more likely to develop a PUR. This probably is attributed to the higher bacillary burden as suggested by several observations.^17^,^18^ Similarly, patients with bilateral extensive adenopathy had 2.9 times greater odds of developing PUR, again likely to the disease burden. Presence of extensive necrosis on histopathology also emerged as a risk factor independently associated with the development of PUR. In addition, anorexia, though a subjective symptom, was associated with PUR development.

Unlike the findings of Cho et al.^9^ where younger age and male gender were associated with PUR, there was no difference in the age, sex and BMI distribution in our case and control groups. Moreover, Cheng et al observed that patients with paradoxical reactions had a significantly lower absolute lymphocyte count at baseline (675+/- 315 cells/micro L), but our cohort did not have baseline lymphopenia.^13^ Another study had found anemia at presentation to be a potential predictor of PUR but this was not borne out in our study.^1^

### Limitations of the study

We excluded drug-resistant (DR) tuberculous lymphadenitis owing to the long follow up and more complex treatment required, therefore our study cannot predict the nature and severity of PUR occurring in DR TB cases. Another limitation is that the short post-treatment follow-up may have missed late-onset post-therapy PUR. Most of our TBLA patients were presumed cases, based on suggestive histopathology rather than confirmed microbiology. Hence, we cannot comment accurately on the contribution of Non Tuberculous Mycobacteria (NTM) which has similar drug treatment as in our presumed TB cases. Further studies need to be done to assess if PUR is associated with NTM as well.

## CONCLUSIONS

We found that PUR was relatively common, occurring in 17.5% of TBLA patients after ATT commencement. The reactions were usually mild and respond well to NSAIDs. Predictive factors of PUR in patients with TBLA were, anorexia, bilateral extensive, lymphadenopathy, the presence of AFB on smear microscopy and extensive necrosis on histopathology.

### Authors’ Contribution

**SS:** Conceived the idea, designed the proposal, writing & editing of manuscript.

**SI:** Statistical analysis, results write-up, formatting & editing of the manuscript.

**NS:** Contributed to final concept and design. Drafted, formatted and then critically edited the manuscript.
